# Supplementary material for: Client preferences in noncommunicable diseases management in Australia: A scoping review
Source: PLOS Glob Public Health. 2025 Dec 3;5(12):e0005568. doi: 10.1371/journal.pgph.0005568 (PMC12674526; doi:10.1371/journal.pgph.0005568)
Supplement: S1 Table — (DOCX) [file pgph.0005568.s002.docx]

S1 Table-Search strategies

| Databases | Search strategies | Search results on 23 Oct 2024 |
| --- | --- | --- |
| PubMed | ("discrete choice experiment*"[Title/Abstract] OR "discrete-choice experiment*"[Title/Abstract]) AND (((((((((((((("noncommunicable disease*"[Title/Abstract]) OR ("non communicable disease*"[Title/Abstract])) OR ("non-communicable disease*"[Title/Abstract])) OR ("chronic disease*"[Title/Abstract])) OR ("chronic lifelong condition*"[Title/Abstract])) OR ("chronic life long condition*"[Title/Abstract])) OR ("chronic life-long condition*"[Title/Abstract])) OR ("Cardiovascular disease*"[Title/Abstract])) OR (cancer*[Title/Abstract])) OR ("chronic respiratory disease*"[Title/Abstract])) OR (COPD[Title/Abstract])) OR (diabet*[Title/Abstract])) OR (mental*[Title/Abstract])) OR (NCD[Title/Abstract])) | 731 |
| Web of Science | (TI=("discrete choice experiment*" OR "discrete-choice experiment*")) AND TI=("noncommunicable disease*" OR "non communicable disease*" OR "non-communicable disease*" OR "chronic disease*" OR "chronic lifelong condition*" OR "chronic life long condition*" OR "chronic life-long condition*" OR "Cardiovascular disease*" OR cancer* OR "chronic respiratory disease*" OR copd OR diabet* OR mental* OR ncd) | 372 |
| Scopus | ( TITLE-ABS-KEY ( "discrete choice experiment*" OR "discrete-choice experiment*" ) ) AND ( TITLE-ABS-KEY ( "noncommunicable disease*" OR "non communicable disease*" OR "non-communicable disease*" OR "chronic disease*" OR "chronic lifelong condition*" OR "chronic life long condition*" OR "chronic life-long condition*" OR "Cardiovascular disease*" OR cancer* OR "chronic respiratory disease*" OR copd OR diabet* OR mental* OR ncd ) | 928 |
| EMBASE | ('discrete choice experiment*':ab,ti OR 'discrete-choice experiment*':ti) AND ('non communicable disease*':ab,ti OR 'noncommunicable disease*':ab,ti OR 'non-communicable disease*':ab,ti OR 'chronic disease*':ab,ti OR 'chronic lifelong condition*':ab,ti OR 'chronic life-long condition*':ab,ti OR 'chronic life long condition*':ab,ti OR 'cardiovascular disease*':ab,ti OR cancer*:ab,ti OR 'chronic respiratory disease*':ab,ti OR copd:ab,ti OR diabet*:ab,ti OR mental*:ab,ti OR ncd:ab,ti) | 1,154 |
