# Supplementary material for: Client preferences in noncommunicable diseases management in Australia: A scoping review
Source: PLOS Glob Public Health. 2025 Dec 3;5(12):e0005568. doi: 10.1371/journal.pgph.0005568 (PMC12674526; doi:10.1371/journal.pgph.0005568)
Supplement: S2 Table — (DOCX) [file pgph.0005568.s003.docx]

S2 Table: Study characteristics regarding the implementation of the DCE

| Authors | Methods to attribute selection and levels | Approach to experimental study design | Types of statistical model | Number of attributes | Participants |
| --- | --- | --- | --- | --- | --- |
| Ahmed A et al 2021 (1) | Literature review and qualitative research | The International Society for Pharmacoeconomics and Outcomes  Research | A mixed multinomial logit | 8 | 141 |
| Bessen T et al 2014 (2) | Literature and expert consultation | A fractional factorial design | A random utility maximisation framework, the random parameter logit model | 5 | 722 |
| Broomfield G et al 2022 (3) | Literature review and qualitative research | Full factorial design | Cumulative link models | 4 | 329 |
| Brown A et al 2022 (4) | Literature review, pilot, expert opinion | A generator-developed design | Multinomial logit modelling and Latent Class Analysis | 6 | 476 |
| Fifer S et al 2018 (5) | Literature review and consultation | A Bayesian D-efficient design | Error Components model | 10 | 171 |
| Fifer SJ et al 2020 (6) | Literature review, qualitative research and expert opinion | Unlabelled design | Latent Class Mode | 6 | 124 people living with multiple myeloma, 44 carers, 28 haematologists, and 34 nurses |
| Fifer S et al 2022 (7) | Qualitative interviews, literature reviews and expert opinion | Bayesian D‐efficient de‐ sign with naïve priors to | A mixed multinomial logit model | 9 | 45 patient and 44 clinicians |
| Goodall S et al 2012 (8) | Literature and FGDs | unlabelled design, Factorial design | A mixed (random effects) logit model | 6 | 83 patients and 78 carers |
| Herrmann A et al 2018 (9) | Literature review and consultation | not reported | Descriptive & correlation | 2 | 157 |
| Hobden B et al 2018 (10) | Consultation | counterbalanced Latin square design. | R prop. Test | 3 | 281 |
| Howard K et al 2014 (11) | Literature and consultation (discussion) | D-efficient fractional factorial design | A mixed logit model | 6 | 662 |
| Howard K et al 2023 (12) | Literature review, interview, and consultation | D-efficient DCE design |  | 8 | 420 |
| Kenny P et al 2024 (13) | Literature review and consultation | separate generator-developed design | mixed logit model | 12 for cancer, 11 for Dementia and 10 for Heart failure | 1548 for cancer, 1549 for dementia, 1003 for heart failure |
| Livingstone A et al 2023 (14) | Literature review and in-depth discussion | D-efficient design | mixed multinomial logit model | 6 | 116 |
| De Abreu Lourenço R et al 2019 (15) | literature review and qualitative research |  | Latent class analysis |  | 464 |
| Nickel B et al 2018 (16) | literature review and qualitative research | Bayesian efficient DCE design | A mixed logit model | 8 | 2054 |
| Salkeld G et al 2005 (17) | Survey and authors decision | Fractional factorial design | A probit model | 4 | 103 |
| Senanayake S et al 2024 (18) | a review of the literature,  focus groups with consumers and health service providers, a  quantitative structured prioritisation exercise, and an expert  panel discussion | Bayesian D-efficient design | a latent class  modelling | 5 | 123 |
| Snoswell CL et al 2018 (19) | Review and pilot study | D-efficient | A mixed logit model | 7 | 113 |
| Spinks J et al 2016 (20) | Literature review | A multinomial logit d-efficient design | A mixed logit mode | 7 | 35 |
| Turon H et al 2020 (21) | Not clear | A fully crossed design | Probit link function | 2 | 275 |
| Venning B et al 2022 (22) | literature review, expert opinion, and consumer focus groups | Generator developed design | mixed logistic regression, latent class analysis | 8 | 1,002 |
| Whitty JA et al 2015 (23) | Literature review and qualitative research | A full factorial design | Mixed logit models | 6 | 602 clients and 297 health professionals |
| Wong SF et al 2016 (24) | literature and an analysis of qualitative research | A smaller fractional factorial design | A mixed logistic regression | 6 | 185 |
| Yim J et al 2021 (25) | Literature review and using the Australian Clinical Pathway for the Screening, Assessment, and Management of anxiety & depression in Adult Cancer Patients, qualitative research | D-efficiency design | conditional logit model and mixed logit model | 8 | 294 |
| Yu A et al 2021 (26) | Literature review and qualitative research | A generator-developed experimental design |  | 6 | 117 |
| Howard K et al 2011 (27) | Literature review, qualitative research and expert opinion | Full factorial design | A mixed logit model | 4 | 130 |
| Liede A et al 2017 (28) | Literature review and consultation with clinical experts | D-optimal algorithm | Random logit model | 7 | 622 |
| Ride J et al 2024 (29) | Literature review, FGD, advocacy group, online carer sessions | Bayesian D-efcient design | A conditional logit model | 3 | 55 |
| Senanayake S et al 2024 (30) | Systematic review of the literature, qualitative interviews, a quantitative structured prioritisation exercise and an expert panel discussion | D-efficient design | A mixed multinomial logit mode | 5 | 305 |
| Venning B et al 2024 (31) | Qualitative research, and pilot testing was conducted of the survey online and with consumers | A D-efficient fractional factorial design | A mixed logit model | 5 | 3,013 |
| Waller A et al 2018 (32) | Consultation and pilot test | Not clearly mentioned | A probit link function | 3 | 110 patients and 64 support persons |

**References**

1. Ahmad A, Khan MU, Aslani P. Patient preferences for the treatment of type 2 diabetes in Australia: a discrete choice experiment. Journal of Diabetes & Metabolic Disorders. 2022;21(1):229-40.

2. Bessen T, Chen G, Street J, Eliott J, Karnon J, Keefe D, et al. What sort of follow-up services would Australian breast cancer survivors prefer if we could no longer offer long-term specialist-based care? A discrete choice experiment. British journal of cancer. 2014;110(4):859-67.

3. Broomfield G, Brown SD, Yap MB. Socioeconomic factors and parents' preferences for internet-and mobile-based parenting interventions to prevent youth mental health problems: a discrete choice experiment. Internet Interventions. 2022;28:100522.

4. Brown A, Pain T, Tan A, Anable L, Callander E, Watt K, et al. Men’s preferences for image-guidance in prostate radiation therapy: A discrete choice experiment. Radiotherapy and Oncology. 2022;167:49-56.

5. Fifer S, Rose J, Hamrosi KK, Swain D. Valuing injection frequency and other attributes of type 2 diabetes treatments in Australia: a discrete choice experiment. BMC health services research. 2018;18:1-11.

6. Fifer SJ, Ho K-A, Lybrand S, Axford LJ, Roach S. Alignment of preferences in the treatment of multiple myeloma–a discrete choice experiment of patient, carer, physician, and nurse preferences. BMC cancer. 2020;20:1-11.

7. Fifer S, Ordman R, Briggs L, Cowley A. Patient and clinician preferences for genetic and genomic testing in non-small cell lung cancer: a discrete choice experiment. Journal of Personalized Medicine. 2022;12(6):879.

8. Goodall S, King M, Ewing J, Smith N, Kenny P. Preferences for support services among adolescents and young adults with cancer or a blood disorder: A discrete choice experiment. Health Policy. 2012;107(2-3):304-11.

9. Herrmann A, Sanson-Fisher R, Hall A, Wall L, Zdenkowski N, Waller A. A discrete choice experiment to assess cancer patients’ preferences for when and how to make treatment decisions. Supportive care in cancer. 2018;26:1215-20.

10. Hobden B, Turon H, Bryant J, Wall L, Brown S, Sanson‐Fisher R. Oncology patient preferences for depression care: a discrete choice experiment. Psycho‐oncology. 2019;28(4):807-14.

11. Howard K, Salkeld GP, Patel MI, Mann GJ, Pignone MP. Men's preferences and trade‐offs for prostate cancer screening: a discrete choice experiment. Health Expectations. 2015;18(6):3123-35.

12. Howard K, Norris S, Salisbury A, Pearce A, Hay L, Stapleton B, et al. Women's Preferences for Hypofractionated Radiation Therapy for Treatment of Early-Stage Breast Cancer: A Discrete Choice Experiment. International Journal of Radiation Oncology* Biology* Physics. 2024;119(1):172-84.

13. Kenny P, Street DJ, Hall J, Agar MR, Phillips J. Community Preferences for the Care of Older People at the End of Life: How Important is the Disease Context? The Patient-Patient-Centered Outcomes Research. 2024:1-13.

14. Livingstone A, Howard K, Menzies AM, Long GV, Stockler MR, Morton RL. Preferences for Adjuvant Immunotherapy in Adults with Resected Stage III Melanoma—A Discrete Choice Experiment. The Patient-Patient-Centered Outcomes Research. 2023;16(5):497-513.

15. De Abreu Lourenço R, Haas M, Hall J, Parish K, Stuart D, Viney R. My mind is made up: cancer concern and women’s preferences for contralateral prophylactic mastectomy. European Journal of Cancer Care. 2019;28(4):e13058.

16. Nickel B, Howard K, Brito JP, Barratt A, Moynihan R, McCaffery K. Association of preferences for papillary thyroid cancer treatment with disease terminology: a discrete choice experiment. JAMA Otolaryngology–Head & Neck Surgery. 2018;144(10):887-96.

17. Salkeld G, Solomon M, Butow P, Short L. Discrete-choice experiment to measure patient preferences for the surgical management of colorectal cancer. Journal of British Surgery. 2005;92(6):742-7.

18. Senanayake S, Kularatna S, Crawford-Williams F, Brain D, Allen M, Hettiarachchi RM, et al. Cancer survivor preferences for breast cancer follow-up care: a discrete choice experiment. Journal of Cancer Survivorship. 2024:1-8.

19. Snoswell CL, Whitty JA, Caffery LJ, Loescher LJ, Gillespie N, Janda M. Direct-to-consumer mobile teledermoscopy for skin cancer screening: Preliminary results demonstrating willingness-to-pay in Australia. Journal of telemedicine and telecare. 2018;24(10):683-9.

20. Spinks J, Janda M, Soyer HP, Whitty JA. Consumer preferences for teledermoscopy screening to detect melanoma early. Journal of telemedicine and telecare. 2016;22(1):39-46.

21. Turon H, Wall L, Fakes K, Brown SD, Sanson-Fisher R. Cancer patient preferences for the provision of information regarding emotional concerns in relation to medical procedures: A discrete choice experiment. Patient Education and Counseling. 2020;103(7):1439-43.

22. Venning B, Saya S, Lourenco RDA, Street DJ, Emery JD. Preferences for a polygenic test to estimate cancer risk in a general Australian population. Genetics in Medicine. 2022;24(10):2144-54.

23. Whitty JA, Kendall E, Sav A, Kelly F, McMillan SS, King MA, et al. Preferences for the delivery of community pharmacy services to help manage chronic conditions. Research in Social and Administrative Pharmacy. 2015;11(2):197-215.

24. Wong SF, Norman R, Dunning TL, Ashley DM, Khasraw M, Hayes TM, et al. A discrete choice experiment to examine the preferences of patients with cancer and their willingness to pay for different types of health care appointments. Journal of the National Comprehensive Cancer Network. 2016;14(3):311-9.

25. Yim J, Arora S, Shaw J, Street DJ, Pearce A, Viney R. Patient preferences for anxiety and depression screening in cancer care: A discrete choice experiment. Value in Health. 2021;24(12):1835-44.

26. Yu A, Street D, Viney R, Goodall S, Pearce A, Haywood P, et al. Clinical assessment of chemotherapy-induced peripheral neuropathy: a discrete choice experiment of patient preferences. Supportive Care in Cancer. 2021;29:6379-87.

27. Howard K, Salkeld G, Pignone M, Hewett P, Cheung P, Olsen J, et al. Preferences for CT colonography and colonoscopy as diagnostic tests for colorectal cancer: a discrete choice experiment. Value in Health. 2011;14(8):1146-52.

28. Liede A, Mansfield CA, Metcalfe KA, Price MA, Cancer KCFCfRiFB, Snyder C, et al. Preferences for breast cancer risk reduction among BRCA1/BRCA2 mutation carriers: a discrete-choice experiment. Breast cancer research and treatment. 2017;165:433-44.

29. Ride J, Cameron L, Jones R, Hall T, Cameron F, White M. Participation and psychosocial supports in the school setting for children with type 1 diabetes: A discrete choice experiment of carer priority. Diabetes Research and Clinical Practice. 2024:111753.

30. Senanayake S, Barnett A, Brain D, Allen M, Powell E, O'Beirne J, et al. A discrete choice experiment to elicit preferences for a chronic disease screening programme in Queensland, Australia. Public Health. 2024;228:105-11.

31. Venning B, Pearce A, Lourenco RDA, Hall R, Bergin RJ, Lee A, et al. Patient preferences for investigating cancer-related symptoms in Australian general practice: a discrete-choice experiment. British Journal of General Practice. 2024;74(745):e517-e26.

32. Waller A, Sanson-Fisher R, Brown SD, Wall L, Walsh J. Quality versus quantity in end-of-life choices of cancer patients and support persons: a discrete choice experiment. Supportive Care in Cancer. 2018;26:3593-9.
