# Supplementary material for: Client preferences in noncommunicable diseases management in Australia: A scoping review
Source: PLOS Glob Public Health. 2025 Dec 3;5(12):e0005568. doi: 10.1371/journal.pgph.0005568 (PMC12674526; doi:10.1371/journal.pgph.0005568)
Supplement: Attachment — Submitted filename: Response to Reviewers.docx [file pgph.0005568.s005.docx]

**Response to Reviewers**

**Manuscript ID: PGPH-D-25-01773
Title: Client Preferences in Noncommunicable Diseases Management in Australia: A Scoping Review**
Dear Academic Editor and Reviewers,

We thank you for your time in managing the review process and providing crucial comments. We have submitted the revised submission in accordance with the PLOS Global Public Health requirements. In our revised submission, we have addressed the comments and submitted the manuscript for publication. The reviewers’ comments are numbered, and our responses are underlined and in *Italic* type.

**Review Comments to the Author**

**Reviewer #1**

1. The article did not describe the ages of those who preferred in person visits. It did not differentiate between older and younger populations and their preference for telemedicine. Did the older patients with NCDs have access to phones and know how to do telemedicine or was it a select group that did telemedicine amongst the NCD population?

*Authors’ response: We appreciate this feedback. Adults are the study participants in the included studies, which assessed the preference to the whole group. However, we added more points in the discussion section to clarify age related matters for mHealth preference based on priori evidence on another area or in another country. We added the following around the end of the fourth paragraph of the discussion section: This implies that mhealth care and face-to-face service preferences may depend on the health disorder conditions, the need for physical examinations, other conditions that require physical presence in a health care setting, and social determinants of health. For example, older adults faced technical barrier, including digital skills in telemedicine care model, according to another review. The National Survey of older US adults on willingness to use telehealth revealed that willingness to use telehealth declines with increasing age, but a lower cost or an insurance coverage and perceived usefulness modify their willingness to use it. Australian Communication and Media Authority highlighted the importance of supporting older people’s digital literacy and providing them with the skills to navigate what can be confusing and potentially risky environments to help them use the internet and engage with the digital world.*

**Reviewer #2**

1. This is an important article that engages with the suitability of models of primary healthcare for specific NCD management and identifies areas for improvement within the Australian context. While this is based on the synthesis of papers on chronic conditions, it could benefit from further clarification.

*Authors’ response: Thank you for the complement and we have addressed the comments in the revised submission.*

2. All the included papers were written during the second decade of the 21st century. This paper, though, mentions chronic care models in Australia. However, it could highlight the kinds of DSD programmes available for the NCDs to give a backdrop to the NCD management.

*Authors’ response: We have briefly highlighted in the method sections of the revised manuscript where DSD is explained. We have included “Although there is limited evidence of the DSD model being explicitly named and applied by listing its domains, healthcare services have been provided through various health professionals across different settings based on client preferences. Several programs for NCD care align with DSD model. For instance, the Integrated Virtual Diabetes Care Clinics in Western Sydney, Australia, offer different service options based on patient complexity, using a combination of virtual and in-person consultations to tailor care according to patient needs and risk levels”.*

3. Page 4 Under Eligibility Criteria,
   “We included preference articles used discrete choice experiment (DCE) among clients with major NCD in general or specifically on cardiovascular diseases (CVD), cancer, chronic obstructive pulmonary diseases (COPD), diabetes, or mental health disorders”. – Out of 32 included papers, the authors can specify how many are under each NCD marked here?

*Authors’ response: We have added one article, which was missed from citation, but was found eligible, and now they are 33 articles included. We have already presented this in the Result Section as follows: “A total of 33 articles was included in the current review after screening of 3,185 articles (Figure 1). Two studies focused on general NCD. Twenty-four studies were on cancer: non-specific types of cancer; breast cancer; skin cancer (melanoma); prostate cancer; colorectal cancer; cancer or blood disorders; multiple myeloma; lung cancer; papillary thyroid cancer; and oesophagogastric, bowel, or lung cancer. Three studies were on DM: type 1 DM and type 2 DM. Three studies addressed mental health: mental health prevention and depression and anxiety. One study focused on dementia, heart failure, and cancer.”*

4. The analysis can benefit from additional inputs and enhance its richness. In the analysis section, disaggregating the 'client' can be helpful in understanding how NCD services are experienced across different demographics, including sex and gender, ethnicity, nationality, and region.

*Authors’ response: We have rereviewed the included studies and extracted social determinants of health influences on clients preference if the articles have reported it. We have included the following in the result section of the revised submission, with citation when necessary:*

*Social determinants of health*

*Social determinants of health influence client’s preference across the continuum of NCD care. These determinants include place of resident, occupation, gender, education status, socioeconomic status, and age. Rural participants preferred shorter external beam radiation therapy and were less willing to extend treatment to reduce toxicity risk. Individuals employed fulltime were less likely to prefer full surgery than not employed fulltime. Differences in preferences between males and females were reported. In cancer-related care and support, males valued assistance with reintegration into work or study, emotional support for their families, and less emotional support for themselves. They placed lower value on culturally specific support compared to females. Males favoured specialist care and preferred to be accompanied by family or friends during cancer treatment. They were also more likely to choose a self-directed approach and least preferred clinician-directed referrals, whereas the opposite was true for females. Females showed a lower preference for melanoma immunotherapy than males.*

*Education level and socioeconomic status influence clients’ care preferences. Parents without formal postsecondary education favoured briefer modules and a predefined module order (less user-controlled). Those who completed high school or higher were more likely to prefer computed tomography colonography (CTC). Individuals with a tertiary education preferred more invasive forms of testing for cancers-related symptoms. However, education level did not affect preferences for prostate-specific antigen screening. Health literacy also influenced preferences: those with lower health literacy were less likely to prefer less invasive treatment options. Regarding socioeconomic status, lower-income parents preferred app-based, inexpensive, and shorter programs in health education. In contrast, income level did not affect preferences for prostate-specific antigen screening, and having private health insurance did not significantly influence test preferences.*

*Age of clients was crucial to in informing care models. Increasing age was associated with a lower preference for prostate-specific antigen screening compared to no screening and a lower preference for CTC. Age did not influence oncology patients’ preference for depression care. Another study addressed differences in surgical preferences between younger and older women in breast-cancer. Younger women preferred breast-conserving surgery, while older women were less influenced by the type of surgery chosen. Age and quality of life also influenced care preferences. Younger women with higher quality of life favoured multidisciplinary care teams (including specialists, nurses, and general practitioners) and valued shared survivorship care plans. In contrary, older women with lower quality of life were more concerned about out-of-pocket costs and remained neutral about team composition. Older preferred shorter external beam radiation therapy and were less willing-to-extend treatment to reduce toxicity risk. Those aged ≥60 years preferred their regular GP and more invasive forms of testing for cancer-related symptoms.*

*We have also addressed the social determinants of health in the discussion section in the revised submission.*
6. The paper draws upon the DSD model. In conclusion, while there is generalisation, it may be strengthened by adding some of the management-specificities of the specific NCD, which can be further addressed in the NCD management. Its implications for integrating healthcare at the PHC level can be highlighted.

*Authors’ response: We have revised the conclusion section as well. It is presented as follows “This review highlights that clients prefer NCD care models that are accessible, person-centered, safe, efficient, and effective. Well-trained healthcare providers, self-directed approach (for men) or clinician-directed referrals (for women), and multidisciplinary teams (e.g., preferred by younger women with higher quality of life) are key components of preferred care models. While telehealth and mHealth services are valued for convenience, face-to-face interactions remain essential for conditions requiring physical assessments. Home-based services, such as self-sampling for cervical cancer and mailed bowel cancer test kits, are appreciated for their accessibility and ease of use. This implies that in informing the development of models of care, clients’ individual characteristics may influence the selection of care attributes or elements, as social determinants of health can be linked to aspects such as accessibility, equity, comprehensiveness, continuity of care, effectiveness, cost-effectiveness, and overall quality of care. In addition, the severity of diseases condition, nature of therapy, and social and cultural values are crucial in the development of person-centred model of care. The review reinforces the importance of strengthening PHC-based health systems to meet client preferences and improve NCD management.”*

5. Table 1 can be reorganised NCD wise

*Authors’ response: We appreciate your suggestions. As Table 1 is mainly about characteristics of studies, the column ‘Disease category’ comprises NCD types and each study category is also described in relation to disease category as presented under Search results in the Result section.*

6. In Figure 1 the box for Screening and Included overlap. This may be checked.

*Authors’ response: We have addressed this in the revised submission.*
